# Transcriptional response of spermathecal secretory cells to mating and receipt of seminal fluid proteins in *Aedes aegypti*

**DOI:** 10.1016/j.isci.2026.116301

**Published:** 2026-06-05

**Authors:** Claudia A.S. Wyer, Mehrnaz Afkhami, David A. Ellis, Sylvie Pitcher, I. Alexandra Amaro, Patrick K. Perish, Jake W. Angelico, Emilie M. Gray, Yasir H. Ahmed-Braimah, Mariana F. Wolfner, Laura C. Harrington

**Affiliations:** 1 Department of Entomology, Cornell University, Ithaca, NY 14853, USA; 2 Department of Molecular Biology and Genetics, Cornell University, Ithaca, NY 14853, USA; 3 Ear Institute, University College London, London WC1X 8EE, UK; 4 Department of Biology, Syracuse University, Syracuse, NY 13244, USA

**Keywords:** entomology, molecular biology, cell biology

## Abstract

*Aedes aegypti* mosquitos are major vectors of arboviruses including dengue, yellow fever, Zika, and chikungunya, highlighting the need for innovative control strategies. Females typically mate only once yet store sufficient sperm to produce hundreds of offspring over their lifetime. During mating, males transfer sperm and seminal fluid proteins from the male accessory glands (MAGs) into the female reproductive tract. Sperm rapidly migrate to the spermathecae, where they remain viable, likely supported by spermathecal secretory cells (SSCs). However, the molecular mechanisms underlying long-term sperm maintenance are poorly understood. We used single-nucleus RNA sequencing to characterize cell types within the spermathecae and confirmed SSC populations using RNA fluorescence *in situ* hybridization. We then compared SSC transcriptional responses following natural mating and MAG lysate injection. Natural mating induced a stronger SSC response after 24 h, suggesting that sperm receipt is critical for SSC activation and sperm storage regulation.

## Highlights

• snRNA-seq reveals diverse cell types in *Ae. aegypti* spermathecae
• Spermathecal secretory cells show strong transcriptional responses to mating
• Natural mating induces stronger SSC responses than MAG injection alone
• SSC gene markers have potential as targets for reproductive control

## Graphical abstract

**Figure:**
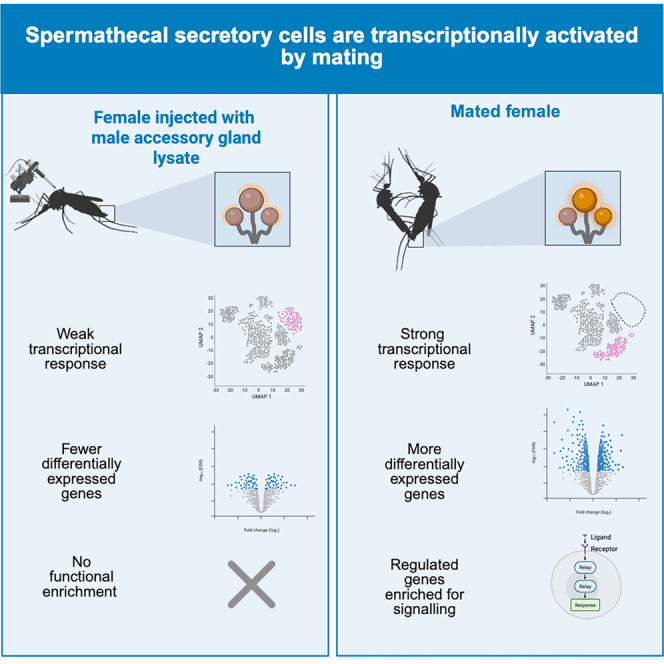

## Introduction

*Aedes aegypti* mosquitoes are a major global threat to public health. This formidable vector transmits viruses with increasing global incidence including dengue, Zika, yellow fever, and chikungunya. Recent dengue outbreaks led to the highest number of dengue cases on record, with over 14 million infections reported worldwide.^1^ Chemical insecticides have played a crucial role in controlling the primary dengue vector, *Ae. aegypti*; however, widespread resistance to the main classes of insecticides presents a major threat to their control and prevention of their associated viruses.^2^^,^^3^ New reproductive control methods present a promising alternative to insecticides for controlling *Ae*. *aegypti* populations.^4^ Reproductive control methods use the mass release of modified male mosquitoes to induce sterility or pathogen resistance in target field populations. Disrupting key components of female fertility presents a promising target for reproductive control. Despite this, there are many aspects of female fertility that are not well understood.

The majority of female *Ae*. *aegypti* mosquitoes mate just once.^5^^,^^6^^,^^7^^,^^8^^,^^9^ From a single insemination, females receive enough sperm for a lifetime of fertilization events.^9^^,^^10^ In addition to sperm, males transfer seminal fluid proteins (SFPs) and other molecules, synthesized mainly in the male accessory glands (MAGs), into the female bursa during copulation.^11^ Less than a minute after copulation, sperm begin to travel up to the spermathecal ducts into the spermathecae where they are stored.^12^^,^^13^ Here, sperm remain viable for the female’s lifetime, likely nourished by the glandular secretions of the spermathecal secretory cells (SSCs), located on the exterior of the spermathecal capsule.^14^^,^^15^ Our understanding of the SSCs in *Ae. aegypti* has previously been limited to morphological observations of cell size and shape. Clements and Potter were the first to describe in detail these principal cell types associated with the spermathecae.^14^ They noted a high ribosome content of the SSCs, suggesting high levels of protein synthesis. Another early study hypothesized that SSC secretions may provide nourishment and/or serve as a chemical attractant for sperm.^16^ Previous work in *Drosophila* demonstrated that SSCs are essential for optimal fertility^17^ and for recruiting sperm to the spermatheca.^18^ This process includes the secretion of extracellular vesicles by the SSCs, a response that is initiated by mating, with the receipt of both sperm and SFPs being essential for normal SSC secretory activity.^19^

Previous studies in *Ae. aegypti* have described a mating-induced transcriptional response of the tissues surrounding the spermathecae^20^^,^^21^ and the female reproductive tract more broadly.^22^ Camargo et al.^20^ identified a host of genes expressed differently in the spermathecae between virgin and mated females at multiple time points after mating. They reported ion transport proteins as among those most significantly upregulated as well as those involved in mitigating the effects of oxidative stress. Camargo et al. performed their study using bulk RNA-seq of dissected *Ae. aegypti* spermathecae and spermathecal ducts. While their results enhanced our understanding of mating-induced changes to the spermathecae, it is not known precisely which cells were driving the broad transcriptional response they observed, and whether or not the response was initiated by the presence of sperm, SFPs, or both.

Here, we used single-nucleus RNA sequencing (snRNA-seq) to profile individual cell types in the *Ae. aegypti* spermathecae. Their distinct gene expression patterns enabled greater resolution of cell types in the spermathecae and the identification of the SSCs, which we validated using *in situ* hybridization of a highly specific biomarker of unknown function. Further, we characterized the transcriptional response of the SSCs to natural mating treatment and injection of SFPs compared with those to virgin and injection of saline treatments, respectively.

## Results

### Nucleus clustering and assigning cell types

**Figure 1:**
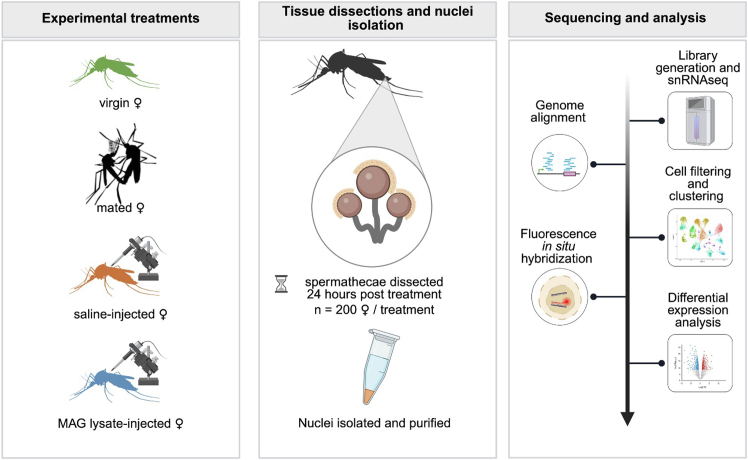
Schematic of the experimental design Spermathecae samples collected from virgin, mated, saline-injected, and MAG lysate-injected female *Ae. aegypti* mosquitoes. Dissections were performed at 24 h post-treatment before subsequent nucleus isolation and snRNA-seq and analysis. Created in https://BioRender.com.

**Figure 2:**
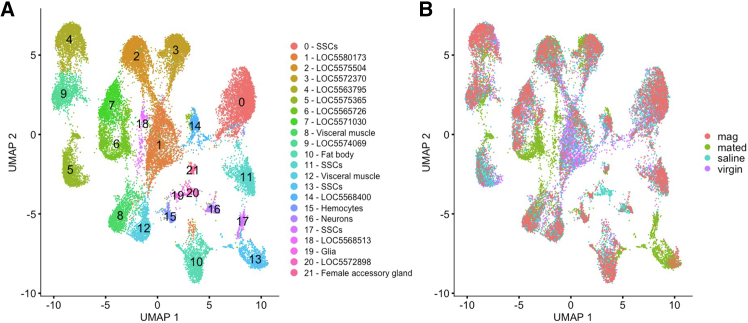
Cell clustering analysis of spermathecal cell types (A and B) UMAP visualization of spermathecal cell clustering across 4 unintegrated treatments: MAG-injected, mated, virgin, and saline-injected females, based on the first 22 principal components, with chosen Seurat resolution of 0.5. (A) Cell clusters are colored and numbered by cell type; in cases where cell type identity is not known, top biomarker gene IDs are given. (B) Cell clusters are colored by treatment.

To determine how the expression profiles of SSCs respond to mating and receipt of MAG proteins in *Ae. aegypti*, we generated four snRNA-seq datasets of spermathecae dissected from 200 of each virgin females, mated females, females injected with saline, and females injected with MAG protein lysate (Figure 1). After quality control filtering to eliminate cells expressing very few genes (<200) and genes expressed in very few cells (<3), we retained 26,985 nuclei: 6,196 from virgin females, 5,048 from mated females, 8,180 from MAG-injected females, and 7,561 from saline-injected females. Though our target was the SSCs, inevitably with manual dissections, nuclei from additional cell types were sequenced in our samples. Therefore, to distinguish between unique cell types, we clustered nuclei based on the first 22 principal components (Figure S1) of gene expression variation and discovered 22 cell clusters (Figure 2A; Figure S2). A uniform manifold approximation and projection (UMAP) plot showed that certain cell clusters were essentially unique to certain treatments; cluster 17, for example, was overwhelmingly populated by cells from the mated treatment (Figure 2).

Annotating distinct cell types in non-model organisms can be challenging due to a lack of established genetic markers. Where possible, we inferred the identity of a number of cell clusters based on the expression of published marker genes in *Ae. aegypti* (Data S1)*.* When *Ae. aegypti* markers were not available, we used information on orthologous genes in the fruit fly *D*. *melanogaster,* identified through NCBI^23^ or VectorBase.^24^

Clusters 8 and 12 were identified as visceral muscle cells based on expression of the established *Ae. aegypti* marker gene *bent* (LOC5580168).^25^ Cluster 10 was identified as fat body cells based on a high expression of *Ae. aegypti* orthologues (LOC5574685 and LOC5568426) of the *D. melanogaster* fat body markers *Adipokinetic hormone receptor orthologue* (FBgn0025595) and *Delta [1]-pyrroline-5-carboxylate synthase orthologue* (FBgn0037146).^26^ Cells in cluster 15 were determined to be hemocytes, based on expression of the *Ae. aegypti* hemocyte markers *NimB2* (LOC110674010) and *LRIM16* (LOC5570883),^25^^,^^27^ as well as expression of orthologues of the *D. melanogaster* hemocyte markers *SPARC*, *CDIP*, and *Papilin* (LOC5578380, LOC5570883, and LOC5572409, respectively). Cluster 16 was identified as a population of neurons due to the expression of published *Ae. aegypti* neuron markers *Synaptonagmin 1* and *Bruchpilot*.^28^ Cells in cluster 19 were assigned as glia cells due to a high expression of an established marker *reversed polarity*.^28^^,^^29^ Though not intentionally included in the tissue dissections, cells in cluster 21 likely represent the secretory cells of the female accessory gland (AGSCs), as they express several genes orthologous to the biomarkers recently identified for the *D. melanogaster* AGSCs.^26^

The known marker for SSCs in *D. melanogaster*, *Send1*, does not have an orthologue in *Ae. aegypti*. However, a recent study by Thayer et al.^26^ generated a list of top marker genes for the SSCs, of which the most significant was FBgn0003975, the transcription factor “vestigial.” The *Ae. aegypti* orthologue of this gene, also named *vestigial* (LOC5573506), was highly expressed in clusters 0, 11, 13, and 17, which were hypothesized to be SSCs. In addition, these clusters expressed the highest number of mRNA transcripts (Figure S3A) from a high diversity of genes (Figure S3B), and a high number of genes that encode proteins with signal peptides, all features that are expected of secretory cells (Figure S4).

**Figure 3:**
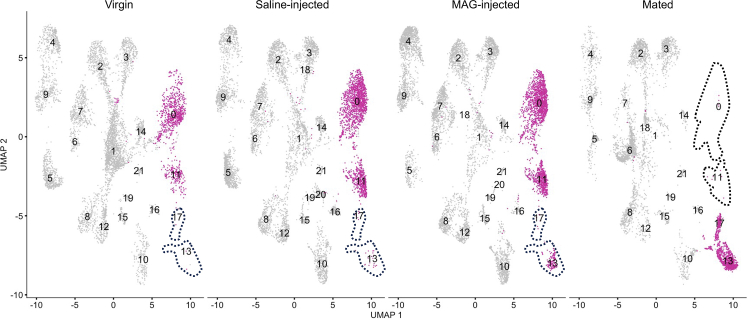
UMAP plots showing spermathecal cell type clusters in *Ae. aegypti* Shown are the four different treatments, with the SSCs highlighted in each. Dotted lines in the mated plot outline the missing cell clusters present in the other three treatments.

To see whether any of the treatments changed the transcriptional landscape of the SSC populations, we separated the treatments, retaining their original embeddings, and plotted them side-by-side (Figure 3). Interestingly, SSC clusters 17 and 13 were almost completely absent from the virgin and saline-injected samples, becoming apparent in MAG-injected and completely dominating the representation of SSCs in the mated sample. In contrast, clusters 0 and 11 were completely absent from the mated sample. This shows the distinct nature of the SSCs after mating.

We further investigated these putative SSC clusters by performing a trajectory analysis. Rather than being distinct and unrelated cell types, the two clusters in each treatment likely represent a continuum between different transcriptional states (Figures S5A and S5B), with the expression of certain genes correlating with the changes in cell state (Figure S5C). Those in the mated samples were found at the opposite end of the continuum from the virgin samples, reflecting their progression to this new state.

#### *In situ* validation of SSC clusters

**Figure 4:**
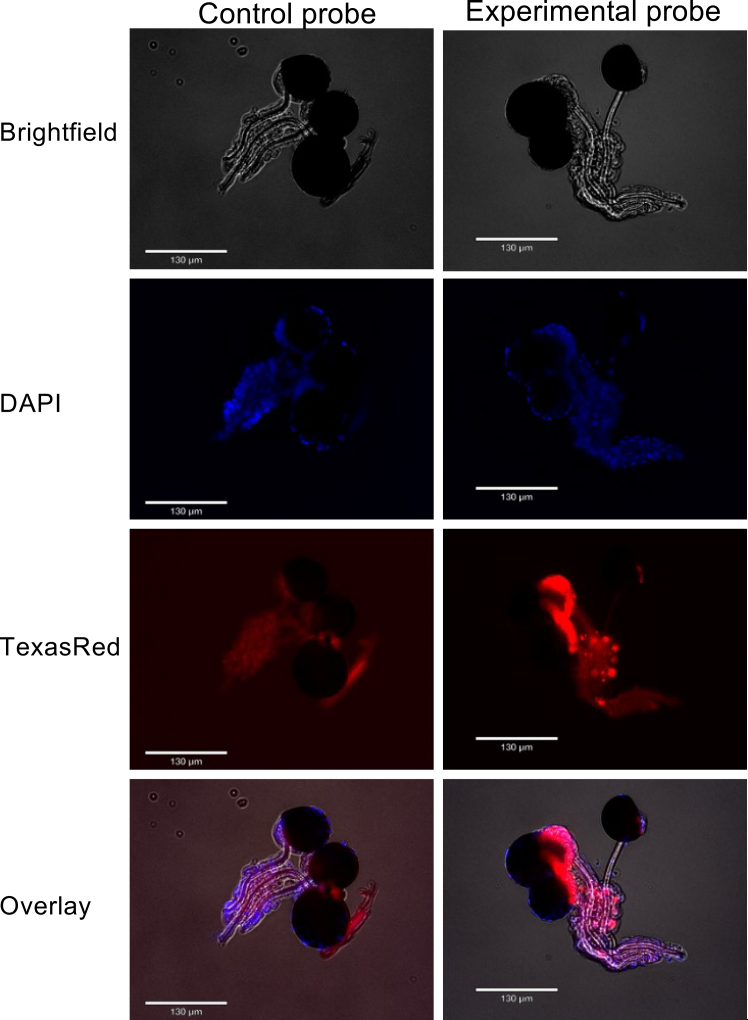
Spermathecal secretory cell marker localization with fluorescence *in situ* hybridization Localization of LOC5577062 in dissected spermathecae and ducts of 3- to 5-day old female *Ae. aegypti* mosquitoes. Scale bars are 130 μm.

To confirm the validity of SSC cluster assignment, we next identified a number of biomarkers and genes expressed with high specificity for these cell clusters (Table S1). RNA fluorescence *in situ* hybridization (FISH) experimental and control probes were designed for LOC5577062 (Table S2), a biomarker gene with relatively high expression in the putative SSCs. Consistent with our snRNA-seq analysis, expression of the biomarker gene LOC5577062 was indeed found to be localized to the SSCs, with low expression in other cells surrounding the spermathecae (Figure 4). We confirmed the absence of signal in the TexasRed channel for the control probe, which is a single-stranded RNA (ssRNA) synthesized with the T7 promotor on the sense strand rather than the antisense strand.

### Differential expression analysis

To understand which genes were driving the difference in transcriptional state between the virgin and mated samples (Figure 3), we first focused on these two treatments for a differential expression analysis. We identified 3,584 significantly differentially expressed genes (DEGs) across all cell types in the dataset (FDR < 0.01, Figure S6). Among these significant DEGs was a putative serine protease (LOC5576159), which expressed in 32.8% of all cells in the virgin treatment but in just 1% of cells in the mated treatment. The general odorant-binding protein 56d (LOC5575341) also showed greater expression in the virgin treatment, with 57.0% of all cells expressing this gene relative to 14.3% of mated cells. Interestingly, a long non-coding RNA (lncRNA) gene of unknown function, LOC110675470, showed the opposite pattern; expression of this gene was the highest in the mated treatment, observed in 67.4% of cells in mated female samples and just 21.9% of cells in virgin female samples (Table 1).

**Table 1:** Top 10 differentially expressed genes in all cells between virgin and mated females

Gene ID	Name	Log_2_FC	*p* value	FDR
LOC110675470	LOC110675470	−2.2026631	<0.001	<0.001
LOC5569692	LOC5569692	−1.4080327	<0.001	<0.001
LOC5573858	zinc carboxypeptidase A 1	1.21589656	<0.001	<0.001
LOC5574378	Nha1 Na[+]/H[+] hydrogen antiporter 1	1.03792173	<0.001	<0.001
LOC5575341	general odorant-binding protein 56d	1.60682652	<0.001	<0.001
LOC5576159	putative serine protease F56F10.1	0.89916498	<0.001	<0.001
LOC5576330	Socs36E suppressor of cytokine signaling at 36E	−1.1894792	<0.001	<0.001
LOC5578454	interleukin 1 receptor accessory protein-like 1	−1.0768826	<0.001	<0.001
LOC5580296	glutamyl aminopeptidase	2.84371709	<0.001	<0.001
LOC5574223	protein 5NUC	−0.5921947	<0.001	<0.001

Positive log fold-change values indicate genes with relatively low expression in mated cells and high expression in virgin cells; negative values indicate genes with low expression in virgin cells and high expression in mated cells.

**Figure 5:**
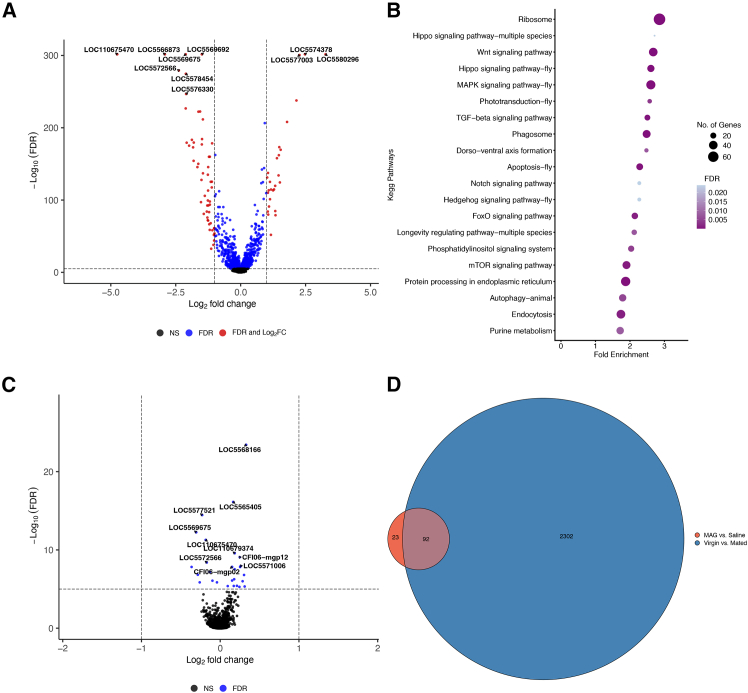
Differential gene expression analysis of spermathecal secretory cells (A) Differentially expressed genes between mated and virgin spermathecal secretory cells (SSCs). Top 10 differentially expressed genes are labeled. Significant genes based on FDR cutoff are denoted by blue dots, and significant genes based on FDR cutoff and log_2_ fold-change greater than 1 are denoted by red dots. FDR and log_2_ fold-change cutoffs are demarcated with dotted lines. (B) Significantly enriched KEGG pathways between virgin and mated SSCs. (C) Differential expression analysis between MAG-injected and saline-injected SSCs. Top 10 differentially expressed genes are labeled. Significant genes based on FDR cutoff are denoted by blue dots. FDR and log_2_ fold-change cutoffs are demarcated with dotted lines. (D) Number of differentially expressed genes between mated and virgin SSCs versus between MAG- and saline-injected SSCs.

Next, we looked at the differential expression of genes expressed in the SSCs (clusters 0,11, 13, and 17, which were combined for this analysis). Interestingly, of the top ten DEGs from the analysis of all cells above, 5 genes (LOC110675470, LOC5569692, LOC5580296, LOC5578454, and LOC5576330) were also represented in the top ten DEGs for the SSCs (Table 2). In total, 2,394 genes were significantly differentially expressed between the mated and virgin SSCs (FDR < 0.01) (Figures 5A and S7A; Data S2).

**Table 2:** Top 10 differentially expressed genes in spermathecal secretory cells between virgin and mated females

Gene ID	Gene name	Log2FC	*p* value	FDR
LOC110675470	LOC110675470	−4.7518121	<0.001	<0.001
LOC5566873	LOC5566873	−2.9176081	<0.001	<0.001
LOC5569675	chico insulin receptor substrate 1 chico	−2.1168215	<0.001	<0.001
LOC5569692	LOC5569692	−1.4954069	<0.001	<0.001
LOC5574378	Nha1 Na[+]/H[+] hydrogen antiporter 1	2.47335705	<0.001	<0.001
LOC5580296	glutamyl aminopeptidase	3.26746271	<0.001	<0.001
LOC5577003	somatostatin receptor type 2	2.26551338	<0.001	<0.001
LOC5572566	NKCC sodium potassium chloride cotransporter	−2.3806125	<0.001	<0.001
LOC5578454	interleukin 1 receptor accessory protein-like 1	−2.1114785	<0.001	<0.001
LOC5576330	Socs36E suppressor of cytokine signaling at 36E	−2.1009816	<0.001	<0.001

Positive values indicate genes with relatively low expression in mated cells and high expression in virgin cells; negative values indicate genes with low expression in virgin cells and high expression in mated cells.

A Kyoto Encyclopedia of Genes and Genomes (KEGG) pathway analysis of the 2,394 significant DEGs (FDR <0.01) in the SSCs compared to all 12,326 genes expressed in all SSCs revealed a 2.86-fold enrichment for genes mapped to ribosomal subunits (Figure 5B). DEGs within the Hippo and Wnt signaling pathways had similarly high fold enrichment scores (2.71 and 2.67, respectively). Functional enrichment analyses revealed that biological processes such as “regulation of signaling,” “peptide metabolic process,” and “regulation of response to stimulus” were all enriched in the set of SSC DEGs (Data S3). Interestingly, it appears that mating also increases the enrichment of genes in the SSCs associated with processes related to egg production such as “oogenesis,” “female gamete generation,” “germ cell development,” and “ovarian follicle cell development.”

In an analysis of DEGs between saline- and MAG-injected female SSCs, we identified just 115 DEGs (FDR < 0.01; Figures 5C and S7B; Data S4). Of those 115 genes, 19 were also differentially expressed between the mated and virgin SSCs (Figure 5D). A KEGG analysis did not yield any functionally enriched pathways, though there was significant functional enrichment for seven biological processes including “morphogenesis of an epithelium” and “establishment or movement of cell polarity” (Data S5).

**Figure 6:**
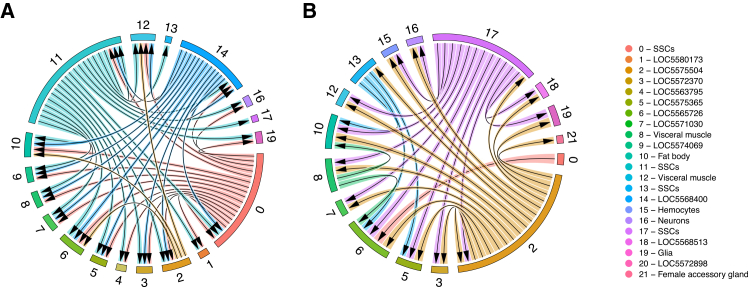
FlyPhoneDB analysis of cell-cell communication in snRNA-seq dataset (A and B) Chord diagram illustrating top scoring, significant interactions between the ligand *dlp* and its receptor *dally*, constituents of the Wnt signaling pathway (*p* < 0.01). (A) Signaling between cell clusters for virgin spermathecae; clusters 0 and 11 represent virgin-specific SSC clusters. (B) Signaling between and within cell clusters for mated spermathecae; clusters 17 and 13 represent mated-specific SSC clusters. Each segment represents a cell cluster, with cluster identity indicated by different colors and numbers on the outer edge of the diagram. Arrows illustrate the direction of the interaction (direction of the interaction is inferred when the interaction score from one cell type to another is higher than the score in the reverse direction).

We used FlyPhoneDB, a database of ligand-receptor pairs that infers cell-cell communication,^30^ to analyze the expression of the 6,264 genes in our dataset with 1:1 orthologue to *D. melanogaster* (46.4% of all genes after filtering) and then predicted ligand-receptor interactions between different cell clusters in both the mated and virgin spermathecae samples. We identified 84 and 79 highly significant ligand-receptor interactions between at least one pair of cell clusters in the virgin and mated treatments, respectively (*p* < 0.01, Data S6). Reiterating the results of the KEGG pathway analysis, we detected enrichment in the Wnt signaling pathway for both treatments, with significant, high scoring interactions between cells expressing the ligand *dlp* (LOC5577324) and cells expressing its receptor *dally* (LOC5567811). In general, one or two clusters were primarily signalers, with multiple others acting as receivers. In the virgin treatment, high scoring, significant interactions were observed between the SSC clusters (0 and 11), cell cluster 14, and multiple other cell types (Figure 6A). Significant ligand-receptor interactions were also observed between cells from the SSC clusters and many cell types in the mated treatment (Figure 6B).

## Discussion

Our results reveal a previously uncharacterized cellular heterogeneity within the spermathecae of the disease vector mosquito *Ae. aegypti* and a pronounced mating-induced transcriptomic response, particularly in SSCs. By grouping cells with similar gene expression patterns, we identified 22 putative cell type clusters in spermathecae under four experimental treatments: virgin, mated, saline-injected, and MAG-injected. We identified cell types that have previously been characterized in *Ae. aegypti*, such as hemocytes,^25^^,^^27^ glial cells, and neurons,^29^^,^^31^ in addition to less well-studied cell types such as the female AGSCs.^26^ We provide several lines of evidence to support the categorization of clusters 0, 11, 13, and 17 as the SSCs. Firstly, by comparing cell types based on their expression using dimensionality reduction methods, we demonstrated that these cell clusters show the strongest response to mating (Figure 3). We also observed that mated SSC clusters 13 and 17 have the highest transcriptional output of all cell types (Figure S3A). Our conclusion is supported by previous work in *D. melanogaster*, which demonstrated that mating increases the secretory activity of the SSCs.^19^ Further, these clusters alone show highly specific expression of the transcription factor “vestigial,” a gene with a transcriptional *D. melanogaster* orthologue that is a biomarker for the SSCs.^26^ To ensure high confidence in the assignment of these clusters as SSCs, we also demonstrated *in situ* that these cells express another biomarker gene for these clusters identified in our analysis (LOC5577062, Figure 4).

Interestingly, for each of the treatments in this study, the SSCs appear to form two distinct cell clusters, with clusters likely representing cells within the same lineage but in different transcriptional states along a continuum (Figure S5). We propose two possible explanations for this observation: firstly, each cell cluster corresponds to secretory cells on different spermathecal capsules. Though *Ae. aegypti* have three spermathecae, they are dimorphic, with one large (100-μm diameter), medial, and two smaller (each 75-μm diameter), lateral spermathecae.^14^^,^^15^ Recent work in *D. melanogaster* demonstrated that the two spermathecae, despite ostensibly similar in morphology, in fact are divergent in terms of form, function, and developmental origin.^32^ It is an exciting possibility that the two distinct clusters of SSCs in our dataset represent developmental or functional asymmetry in these important organs and constitutes a promising avenue for future research. Alternatively, there was a small population of secretory cells along the spermathecal ducts,^14^ which could conceivably have a different expression profile from those on the spermathecal capsules. The source of these distinct subpopulations of secretory cells could be determined with additional *in situ* experiments utilizing cluster-specific marker genes generated in our study.

Identification of genetic markers for the SSCs creates the possibility of developing genetic lines with SSC-specific drivers. For example, driving the expression of pro-apoptotic genes that initiate cell death could help to further our understanding of the role of these cells. A study in *D. melanogaster* showed that genetic ablation of the SSCs resulted in reduced sperm motility in the seminal receptacle and the retention and internal development of fertilized eggs.^18^ Should the SSCs themselves, or the expression of certain genes by the SSCs, be essential for maintaining sperm viability and, therefore, fertility, they could be promising targets for developing tools aimed at reducing the population sizes of *Ae. aegypti.* Functional validation of this hypothesis could be achieved by coupling SSC-specific perturbations with established live/dead sperm viability assays, such as dual SYBR-14 and propidium iodide staining (used previously in insects^33^^,^^34^), to directly quantify the effects on stored sperm integrity.

The results of our differential expression analysis demonstrated that natural mating, i.e., receipt of both sperm and SFPs from a male, triggers large changes in gene expression and affects multiple signaling pathways in the SSCs. While previous studies have described transcriptional changes in the spermathecae^20^ and the female reproductive tract^35^ of *Ae. aegypti* in response to mating, we present the first investigation of SSCs at single-cell resolution. Our data indicate dramatic changes in the expression of genes involved in signaling 24 h after mating (Figure 5B); among those are a number of genes encoding G protein-coupled receptors (GPCRs). In insects, GPCRs regulate a wide array of processes, including physiology,^36^^,^^37^ feeding,^38^ development,^39^ behavior,^40^ and reproduction.^41^^,^^42^^,^^43^ Notably, LOC5566873, a gene whose expression increases more than 7-fold in mated SSCs compared with virgins (Table 2), is predicted, based on sequence, to encode a family of GPCRs, which bind to glutamate. In contrast, LOC557003, which encodes a GPCR that binds to the neuropeptide somatostatin, is robustly downregulated in the SSCs after mating. Consistent with these changes in signaling pathway activity following mating, our ligand-receptor interaction analysis revealed extensive communication between SSCs and other cells types, including interactions involving the heparan sulfate proteoglycan co-receptors *dlp* and *dally* (Figure 6). In *Drosophila*, these molecules are key regulators of the Wingless/Wnt signaling pathway, where they control ligand distribution and facilitate effective receptor-mediated signaling between cells.^44^ Given the established roles of Wnt signaling in regulating tissue homeostasis and intercellular communication, these interactions may contribute to the transcriptional responses observed following mating. We identified many additional ligand-receptor interactions between cells in our dataset (Data S6), which are not explored further here but may represent a useful resource for future studies of SSC function and post-mating responses.

Results of the study by Camargo et al.^20^ suggested that the SSCs may have a role in regulating the pH and osmotic environment in the spermathecae, such that it is suitable for sperm storage. Here, we showed that expression of *Nha1*, a gene with known function in maintaining intracellular pH in eukaryotes,^45^^,^^46^ is significantly downregulated in the SSCs after mating. While some insects maintain a more alkaline pH in the spermathecal lumen relative to the hemolymph to facilitate sperm storage,^47^^,^^48^ the pH of the *Ae. aegypti* spermathecal lumen is unknown and warrants further investigation. Disrupting sperm storage conditions, such as pH, could be useful in developing new reproductive control tools for mosquitoes.

Notably, many more DEGs were detected between virgin and mated SSCs than between saline-injected and MAG-injected SSCs, with DEGs not enriched for any particular biochemical or regulatory pathways identified in the saline vs. MAG KEGG analysis. This result suggests that the receipt of MAG products alone is not sufficient to initiate full transcriptional activity of the SSCs. In a recent study in *D. melanogaster,* females mated to males lacking either sperm or MAGs showed reduced secretory activity of the SSCs compared with females mated to wild-type males,^19^ suggesting that both sperm and MAGs are required for SSC activity. While injection of virgin females with MAG lysate in our experiments did not elicit such a large transcriptional response in the SSCs, MAG products may still have a role in their activation. Although intrathoracic injection with MAG lysate is sufficient to induce post-mating-like responses in virgin female *Ae. aegypti*^49^^,^^50^^,^^51^^,^^52^ and transcriptomic changes in the lower reproductive tract,^35^ it may be the case that molecules produced in the MAGs need to be transferred directly into the female bursa in order to have the intended effect on the transcriptional activity of the SSCs specifically. Further, injecting a lysate of whole MAG tissue, rather than just what would be secreted and transferred in the ejaculate during a natural mating, may potentially interfere with its capacity to elicit local transcriptional changes. It is also conceivable that another cue received by females during a natural mating (e.g., physical, pheromonal, or secretions from a tissue other than the MAGs) could influence SSC activity. Analysis of the transcriptomic profile of the SSCs following mating with mutant males lacking sperm but with functioning MAGs^53^ would help resolve this question.

An alternative explanation for the relatively low transcriptional activity following injection with MAG lysate compared with saline-injected controls is that the receipt of certain MAG proteins simply does not affect the spermathecal transcriptome. Studies in *Drosophila* have shown that female gene expression is relatively unaffected by key SFPs such as ovulin and Acp36DE, suggesting that females are already “molecularly poised” to respond to male-derived proteins, synthesizing gene transcripts necessary to respond to mating during development.^54^^,^^55^^,^^56^ It is also plausible that potential transcriptomic changes in the spermathecae occur sooner after mating or MAG injection compared with those tested here; though rates of polyandry decline over the first 20 h post-mating, the vast majority of females (76%) are refractory to a second mating within just 2 h of the first.^57^

Limitations of our approach to characterizing the transcriptional response of the SSCs to mating and receipt of SFPs should be acknowledged. Firstly, our study used a single time point after experimental treatment (24 h) to compare all samples. While a practical necessity, it does not capture the conceivably major transcriptional changes in the SSCs and neighboring cell types across a reproductive cycle or for the potentially extensive duration of sperm storage, and it should be viewed as a snapshot in time, rather than a representative of the full female response. Secretory activity of *Drosophila* SSCs has been shown to be highly dynamic in the first 72 h post-mating alone.^19^ Secondly, the addition of a treatment where females were mated to males lacking MAGs, i.e., a sperm-only treatment, would further clarify the relationship between sperm, MAG proteins, and the SSCs. Though not the focus of this study, it would also be interesting to explore how blood feeding affects the transcriptional activity of the SSCs, given that, as demonstrated by bulk RNA-seq, blood feeding following mating elicits a stronger transcriptional response than mating alone.^20^

In conclusion, our study provides the first single-nucleus transcriptomic characterization of the spermathecae in *Ae. aegypti*, revealing remarkable cellular diversity, and identifies key transcriptional responses of the SSCs to mating. Our results strongly support the identity of SSC clusters based on transcriptional activity, functional parallels with *D. melanogaster*, and *in situ* expression of cell type-specific biomarkers. We show that natural mating induces substantial gene expression changes in SSCs, implicating them in processes critical to sperm maintenance and egg production, while the contribution of MAG products alone (at least at the time point, concentration, and delivery method tested in this study) appears insufficient to elicit the full SSC response. These findings enhance our understanding of post-mating physiology in mosquitoes and highlight the SSCs as promising targets for disrupting fertility in this major human disease vector. Future studies leveraging the genetic markers identified here will be instrumental in dissecting the precise functional roles of SSCs and their potential for novel vector control interventions.

### Limitations of the study

Several limitations of this study should be considered when interpreting our findings. First, transcriptomic analyses were performed at a single post-treatment time point (24 h), providing only a snapshot of SSC activity and potentially missing important temporal dynamics associated with early post-mating responses. Second, while MAG lysate injection was used to isolate the effects of SFPs, this approach may not fully recapitulate the composition of naturally delivered MAG-derived products during mating. Third, the absence of a sperm-only treatment limited our ability to disentangle the relative contributions of sperm and MAG-derived factors to SSC transcriptional activation.

## Resource availability

### Lead contact

Requests for further information on all experiments conducted as part of this report, as well as for resources, should be directed to the lead contact, Laura C. Harrington (lch27@cornell.edu).

### Materials availability

This study did not generate new unique reagents.

### Data and code availability

Original, unprocessed snRNA-seq data files have been deposited at ENA archives (study accession number: PRJEB110525) and are publicly available as of the date of publication. Any additional information required to reanalyze the data reported in this paper is available from the lead contact upon request.

## Acknowledgments

This work was supported by 10.13039/100000002NIH/10.13039/100000060NIAID grant R01-AI095491 awarded to L.C.H and M.F.W. D.A.E is supported by a Marie Skłodowska Curie Actions Global Postdoctoral Fellowship, underwritten by U.K Research and Innovation (10.13039/100015980EP/Z001994/1: HORIZON-MSCA-2023-PF-01-01). The authors would like to thank Yassi Hafezi and the BRC Genomics Facility (RRID: SCR_021727) at the Cornell Institute of Biotechnology (http://www.biotech.cornell.edu/brc/genomics-facility) for sequencing experiments. We also thank Jean-Paul Paluzzi and Leslie Babonis for helpful guidance regarding the RNA FISH aspect of the project.

## Author contributions

Conceptualization, C.A.S.W., L.C.H., M.F.W., and Y.H.A.-B.; methodology, C.A.S.W., M.A., D.A.E., L.C.H., M.F.W., and Y.H.A.-B.; formal analysis, C.A.S.W.; investigation, C.A.S.W., M.A., D.A.E., I.A.A., S.P., P.K.P., J.W.A., E.M.G., and Y.H.A.-B.; resources, L.C.H., M.F.W., and Y.H.A.-B.; writing, C.A.S.W., L.C.H., M.F.W., and Y.H.A.-B.; funding acquisition, D.A.E., L.C.H., M.F.W., and Y.H.A.-B.; supervision, L.C.H., M.F.W., and Y.H.A.-B.

## Declaration of interests

The authors declare no competing interests.

## STAR★Methods

### Key resources table

**Table:** 

REAGENT or RESOURCE	SOURCE	IDENTIFIER
**Antibodies**		

Mouse anti-DIG biotin-conjugated antibody	Jackson ImmunoResearch Laboratories Inc.	RRID: AB_2339017; cat# 200-062-156

**Experimental models: Organisms/strains**		

*Aedes aegypti* (Thai strain)	–	N/A

**Critical commercial assays**		

Chromium GEM-X Single Cell 3′ v4 Assay	10× Genomics	–
Alexa Fluor 555 Tyramide SuperBoost Kit, streptavidin	ThermoFisher	B40933
Digoxigenin (DIG) RNA labeling mix (10×)	Roche Applied Science	11277073910
Nuclei Isolation Kit: Nuclei PURE Prep	Sigma-Aldrich	NUC201
TURBO DNA-free Kit	Life Technologies	AM1907
Lunascript RT SuperMix	NEB	E3010S
CloneAmp HiFi PCR premix	Takara	639298
Monarch PCR cleanup kit	NEB	T1030
TranscriptAid T7 High Yield Transcription Kit	ThermoFisher	K0441
Qubit Broad Range Assay Kit	ThermoFisher	Q32851

**Chemicals, peptides, and recombinant proteins**		

Schneider’s *Drosophila* medium	Invitrogen	21720024
HEPES (pH = 7.4)	Sigma-Aldrich	H3375
Phosphate-buffered saline	Sigma-Aldrich	P4417
KCl	Sigma-Aldrich	P5405
MgCl_2_.6H_2_0	Fisher	M35-500
NP-40 alternative	Sigma	492016
Sucrose	Sigma-Aldrich	S-0389
BSA	Sigma-Aldrich	B4287
Spermidine	Sigma-Aldrich	S2626
Spermine	Sigma-Aldrich	S3256
Pierce™ Protease Inhibitor Mini Tablet, EDTA-free	ThermoFisher	PIA32955
RNaseIN	Roche	3335399001
Trizol	ThermoFisher	15596026
Dulbecco’s phosphate-buffered saline	ThermoFisher	J61917.K2
paraformaldehyde	ThermoFisher	28908
Tween 20	ThermoFisher	003005
VECTASHIELD Mounting Medium with DAPI	Vector Laboratories	H-1200-10

**Deposited data**		

snRNA-seq data	This paper	ENA: PRJEB110525

**Oligonucleotides**		

LOC5577062 forward 1	This paper	NA
LOC5577062 reverse 1	This paper	NA

**Software and algorithms**		

CellRanger (version 8.0.1)	Zheng et al.^58^	–
R (version 4.3.3)	–	–
Seurat (version 5.1.0)	Hao et al.^59^	–
SignalP (version 6.0)	Teufel et al.^60^	–
FlyPhoneDB	Liu et al.^30^	https://github.com/liuyifang/FlyPhoneDB
Slingshot	Street et al.^61^	–
ShinyGO (version 0.82)	Ge et al.^62^	http://bioinformatics.sdstate.edu/go/
MAST	Finak et al.^63^	https://rglab.github.io/MAST/
Benchling	Benchling^64^	https://www.benchling.com/

### Experimental model and study participant details

#### Mosquitoes

*Ae. aegypti* mosquitoes originating from Kamphaeng Phet Province (KPP), Thailand, hereafter referred to as ‘Thai’ strain, were reared in an environmental chamber at 71.9% ± 9.5% RH, 28 °C, and under a photo regime of 10 h light, 10 h dark, and 2 h simulated dawn/dusk. Mosquitoes were reared to obtain medium sized adults following our published methods (200 larvae/1 L water, 8 fish pellets^65^). Pupae were sorted by sex and females transferred to small tubes for eclosion, after which the adults were held separately by sex in 4 L bucket cages and provided 10% sucrose *ad libitum*.

### Method details

#### Preparation of male accessory gland (MAG) lysate

Injection of MAG contents into the hemolymph via the thorax is known to induce a gene expression response in the lower reproductive tract of female *Ae. aegypti*.^35^ We used injection of MAG lysate to explore how receipt of these molecules affects gene expression in the SSCs. MAG lysate was prepared as described previously.^35^^,^^66^ Briefly, 100 one-to two-day-old Thai *Ae. aegypti* males were anesthetized on ice and their accessory glands dissected in modified phosphate-buffered saline (137 mM NaCl, 2.7 mM KCl, 10 mM Na_2_HPO_4_, 3 mM KH_2_PO_4_, 2 mM CaCl_2_ pH 7.0) at a ratio of one accessory gland pair per microliter of buffer. Tissue was homogenized with a motorized pestle for ten 1 s pulses, followed by sonication in a BIORUPTOR (Diagenode, Denville, NJ) at 4 °C on “high” setting with a cycle of 15 s on, 15 s rest, 15 s on. After centrifugation at 25,000 g for 30 min at 4 °C, the supernatant was recovered, protein concentration quantified and snap frozen in liquid N_2_ prior to storage at −80 °C.

#### Sample preparation

Approximately 250 female Thai *Ae. aegypti* mosquitoes from four treatment groups were prepared: virgin, saline-injected, MAG-injected, and mated to a Thai male. MAG homogenate was prepared as described above. For the MAG-injected and saline-injected treatments, two-day old females were anesthetized on ice and injected intrathoracically with 250 nL of either MAG homogenate (concentration 1 μg/μL- 0.25 male accessory gland equivalents, approximately the amount transferred to females during a natural mating^50^^,^^67^), or PBS using a Nanoject III (Drummond, Broomall, PA) automatic injector. After injection, females were allowed to recover for 24 h in a 0.5 L cup with 10% sucrose provided *ad libitum*. For the mated treatment, 25 two-day old virgin females were added to a 5 L bucket of 50 two-day old virgin males. A total of 10 replicate buckets were set up at this same 1:2 ratio. Males and females were allowed to mate for 2 h, after which time all females were removed using an aspirator and pooled in a holding cage. After 24 h, females from all four treatments were anesthetized on ice and their spermathecae dissected on a clean microscope slide using fine forceps and Schneider’s *Drosophila* medium (Invitrogen). Spermathecal ducts were removed as much as possible to exclude duct epithelial cells and duct gland cells. For the mated treatment, any spermathecae without stored sperm were discarded. The dissected spermathecae were transferred using a minutien pin to a Dounce homogenizer containing 200 μL of ice-cold Schneider’s *Drosophila* medium. Females were dissected until a pool of 200 tissues per treatment was obtained.

Spermathecal nuclear isolation was conducted following the methods of Gupta and Lazzaro^68^ for isolating fat body nuclei in *Drosophila* as follows. After dissections, the Dounce homogenizer tubes containing 200 spermathecae per treatment were placed in a 1.5 mL microcentrifuge tube and spun at 2500 g for 10 min at 4 °C to collect all the tissues at the base of the tube. The Schneider’s *Drosophila* medium was carefully removed from the tube and 250 μL of ice-cold hypotonic isolation buffer (HIB: 10 mM HEPES pH 7.4, 10 mM KCl, 2.5 mM MgCl_2_.6H_2_0, 0.5 mM spermidine, 0.15 mM spermine, 1 Pierce Protease Inhibitor Mini Tablet per 10 mL buffer, 0.2 U/μL RNaseIN) added, incubating for 5 min to allow the cells to swell. The tissues were then gently homogenized in the buffer with 20 turns of the Dounce pestle.

To minimize loss of nuclei, samples were subsequently handled using pipette tips and LoBind microcentrifuge tubes (Eppendorf) both coated with a pre-blocking solution (1 × PBS (pH = 7.(4) and 1% BSA (Sigma)). The lysate was transferred to a 1.5 mL microcentrifuge tube, with the Dounce homogenizer rinsed in HIB. All centrifugation steps were performed using a swinging bucket rotor with an acceleration rate of 5 and deceleration rate of 5 to avoid sample disturbance. The lysate was spun at 800 g for 7 min at 4 °C. The supernatant was removed and discarded, taking care not to disturb the pellet. The pellet was resuspended in 500 μL hypotonic sucrose buffer (HSB: 10 mM HEPES (pH 7.4), 10 mM KCl, 2.5 mM MgCl_2_.6H_2_0, 0.01% NP-40 alternative (Merck), 0.3 M sucrose, 1 Pierce Protease Inhibitor Mini Tablet per 10 mL buffer, 0.2 U/μL RNaseIN) and centrifuged at 2500 g for 10 min at 4 °C. The supernatant was removed and the pellet resuspended in 500 μL of wash and suspension buffer (WSB: 1 × PBS, 2% BSA (Sigma), 1 Pierce Protease Inhibitor Mini Table per 10 mL buffer, 0.2 U/μL RNaseIN).

#### Nuclei purification

The nuclear suspension was further purified to remove cellular debris by sucrose gradient centrifugation using the Nuclei Isolation Kit: Nuclei PURE Prep (Sigma #NUC201-KT). The Sucrose Cushion Buffer I (SCB I) was prepared by mixing 2.7 mL Nuclei PURE 2 M sucrose cushion solution (component of Sigma #NUC201-KT) and 300 μL Nuclei PURE sucrose cushion buffer (component of Sigma #NUC201-KT) and stored on ice. To a new 15 mL centrifuge tube 500 μL of SCB I was transferred and kept on ice. 900 μL of SCB I was added to 500 μL of nuclei suspension and mixed thoroughly and gently using a pipette. This nuclei suspension mixed with SCB I was overlayed onto the previously aliquoted 500 μL of SCB I, taking care not to mix the two layers. The sample tubes were transferred to a pre-chilled 4 °C centrifuge. The samples were spun at 3500 g for 20 min at 4 °C, with an acceleration and deceleration rate of 5. The tubes were removed and placed on ice. Taking care not to disturb the pellet, 1950 μL of the supernatant was removed, leaving 50 μL in the tube. The pellet was resuspended in 450 μL WSB such that the new volume was 500 μL, and the entire sample was passed through a Flowmi® cell strainer (Sigma) with 40 μM pore size into a 1.5 mL microcentrifuge tube. The sample was concentrated by centrifugation at 800*g* for 10 m with an acceleration and deceleration rate of 5. Taking care not to disturb the nuclei pellet, 450 μL of supernatant was removed, and the tube gently flicked to resuspend the nuclei in the remaining liquid.

#### Library generation and sequencing

Single cell suspensions were run on a Chromium X instrument and libraries were prepared following the Chromium GEM-X Single Cell 3′ v4 Assay (10× Genomics, user guide CG000731, RevA) by the Cornell BRC Genomics Facility (RRID:SCR_021727). We targeted 8–10,000 cells and used 13 cycles of cDNA amplification. Sample quality was confirmed using a Qubit (DNA HS kit; ThermoFisher) to determine concentration and a Fragment Analyzer (Agilent) to confirm fragment size integrity. Libraries were sequenced on a NovaSeqX, 25B flowcell with 2 × 150bp read length.

#### snRNA-seq data preparation, filtering and clustering analysis

For alignment, a custom genome index file was created in CellRanger (version 8.0.(1)^58^ using the ‘mkref’ command and the latest version of the *Ae. aegypti* reference genome.^69^ Reads were aligned to the genome index file, barcodes removed and unique molecular identifier (UMI) counts generated using the ‘count’ command in CellRanger. Raw feature matrix files generated by CellRanger ‘count’ for each treatment were loaded into R (version 4.3.3.) using Seurat (version 5.1.0).^59^ Count matrices were filtered to include only genes present in a minimum of three nuclei, and only nuclei that express a minimum of 200 genes and maximum of 2500 genes. Count matrices for each treatment were normalized using the ‘NormalizeData’ function with a scale factor of 10,000 and scaled with the ‘ScaleData’ function. Count matrices from the four treatment datasets were then combined using the ‘merge’ function.

In order to determine the appropriate number of principal components (PCs) to include in the downstream analysis, a JackStraw analysis^70^ for PCA significance was performed using a randomly subsampled set of 1000 nuclei. Each significant PC was included in the analysis, up to the first non-significant PC. Nuclei were then clustered using the ‘FindNeighbors’, ‘FindClusters’ and ‘RunUmap’ functions using only significant PCs and a resolution value of 0.5.

Marker genes for each cluster were generated using the ‘FindAllMarkers’ function in Seurat. For many clusters, cell type was assigned based expression of published marker genes for *Ae. aegypti*, or orthology to published marker genes for *D. melanogaster*.

#### Signal peptide enrichment analysis

Signal peptides (SPs) are short amino acid sequences located at the N-terminus of proteins that allow for protein secretion and translocation in all organisms. Since the expected function of the SSCs is protein secretion, we might expect enrichment for expression of genes with an SP. SPs can be predicted from protein sequence data using the program SignalP (version 6.0).^60^ A FASTA file of all protein sequences in the *Ae. aegypti* genome was downloaded from the NCBI database (https://www.ncbi.nlm.nih.gov/datasets/genome/GCF_002204515.2/) and run through SignalP. Gene names from a list of all proteins with a peptide sequence were extracted using the ‘ID-mapping’ function in the UniProt database. The total number of genes expressed that were established to contain an SP was calculated and plotted on a UMAP of cell clusters.

#### Cell lineage and pseudotime inference analysis

Single-cell data can provide the resolution to distinguish between closely-related populations of cells. We used the program Slingshot^61^ to further understand how cell types are related and how they transition between different states. We used a subset of the filtered and clustered dataset generated as described above which retained just the SSC clusters from the virgin treatment and the SSC clusters from the mated treatment. To capture the main sources of variation within the reduced dataset, a PCA was performed on the 500 most highly variable genes using the ‘vst’ method in Seurat, then converted into a ‘SingleCellExperiment’ object for trajectory analysis using Slingshot. Slingshot is designed to work optimally with reduced dimensional data, so PCA embeddings from the first four PCs only were used. Trajectory inference was performed using the manually assigned cluster labels to guide lineage inference. This approach enabled reconstruction of transcriptional state pseudotime trajectories, likely reflecting differentiation and mating status progressions.

To identify genes whose expression changed dynamically along the inferred pseudotime, we extracted pseudotime values generated by Slingshot and modeled gene expression as a smooth function of pseudotime using a generalized additive model (GAM) with loess smoothing. The 50 most significant genes, as determined by the model, were inferred to be the most dynamically regulated over pseudotime.

#### Cell-cell communication analysis

Individual cell gene expression is influenced by signals produced by other cells in their environment. Recent work has produced a database of high confidence ligand-receptor pairs for major signaling pathways in *D. melanogaster*.^30^ We used this database and the FlyPhoneDB interface to infer cell-cell communication pathways in our filtered snRNA-seq dataset based on expression of ligand-receptor pairs orthologous to those of *D. melanogaster*. First, we identified *D. melanogaster* orthologues for all genes in our filtered snRNAseq dataset using the ‘convert orthologs’ function from the program ‘orthogene’.^71^ Only genes with 1:1 orthologues were retained for downstream analysis. A matrix of cell UMIs and their *D. melanogaster* orthologue gene names and a data frame of cell UMIs and their assigned clusters were used to run the FlyPhoneDB pipeline locally using the R script downloaded from GitHub (https://github.com/liuyifang/FlyPhoneDB).^30^

#### Differential expression analysis

The statistical framework MAST^63^ was used to identify genes with expression heterogeneity in the SSCs between experimental treatments. This method employs a hurdle model which accounts for variation in the fraction of genes expressed in a cell (cellular detection rate) that is generated by both technical and biological factors.

To assess enrichment of specific gene pathways and ontologies, a KEGG pathway analysis and a gene ontology (GO) terms analysis, respectively, were conducted in ShinyGO (version 0.82: http://bioinformatics.sdstate.edu/go/) using the latest version of the *Ae. aegypti* genome assembly.^69^ Both analyses were run on a list of significantly differentially expressed genes in the SSCs with a false discovery rate (FDR) < 0.01 compared to a list of all genes expressed in the SSCs.

#### Fluorescence *in situ* hybridization

Total RNA was extracted from 50 3–5-day old virgin female spermathecae using Trizol (ThermoFisher), and residual genomic DNA (gDNA) removed using TURBO DNA-free Kit (Life Technologies). Complementary DNA (cDNA) was synthesized from the extracted RNA using the Lunascript RT SuperMix (NEB). Gene-specific primers were designed using Benchling^64^ on mRNA sequence imported in sense orientation (Table S2) and used with CloneAmp HiFi PCR premix (Takara) to amplify target region from cDNA. Amplification was performed on a C1000 thermocycler (Bio-Rad) using the following conditions: 1 min at 98 °C, followed by 35 cycles of 10 s at 98 °C, 10 s at 63.1 °C, 30 s at 72 °C, and lastly a 2 min extension time at 72 °C. The PCR product was purified using Monarch PCR cleanup kit (NEB), and the sequence verified by Sanger sequencing (Cornell BRC, https://www.biotech.cornell.edu/core-facilities-brc/services/dna-sequencing-sanger). The PCR product was then reamplified with the same cycling conditions but instead using primers with the addition of the T7 sequence (5′-TAATACGACTCACTATAGGG-3′) on either the 5′ end of the forward primer (control) or on the 5′ end of the reverse strand using modified primers (Integrated DNA Technologies) (Table S2). cDNA templates were purified using the Monarch PCR cleanup kit (NEB) and 500 ng used to synthesize digoxigenin (DIG)-labeled sense and anti-sense RNA probes using the TranscriptAid T7 High Yield Transcription Kit (ThermoFisher) and substituting the nucleotides for 2 μL of the DIG RNA Labeling Mix (Roche). The reaction mix was incubated at 37 °C for 5 h. DIG-labeled single-stranded RNA (ssRNA) was ethanol precipitated and quantified using the Qubit Broad Range Assay Kit and Qubit 4 Fluorometer (ThermoFisher). To determine integrity, RNA probes were run on a 1% agarose gel using the 2× RNA Loading Dye and the RiboRuler High Range RNA Ladder (ThermoFisher).

Localization of target genes in the spermathecae and associated cells was established with fluorescence *in situ* hybridization (FISH), based on the method described by Sajadi and Paluzzi.^72^ Briefly, 3–5-day old female *Ae. aegypti* were anesthetized on wet ice and their spermathecae dissected in Dulbecco’s phosphate-buffered saline (DPBS) using fine forceps and minutien pins treated with RNaseZAP (ThermoFisher). Approximately 20 spermathecae were dissected into a microcentrifuge tube containing 4% paraformaldehyde (PFA) in DPBS and fixed for 1 h at room temperature. The subsequent steps of the protocol followed that of Sajadi and Paluzzi,^72^ with a few key modifications: namely, tissues were subject to an extended quenching step with 3% H_2_O_2_ for 1 h, to quench endogenous peroxidase activity. For all pre-hybridization and hybridization steps, samples were incubated at 50 °C, and DIG-labeled RNA probes were denatured by incubating at 80 °C for 5 min then applied at a concentration of 4 ng/μL. Prior to antibody washes, samples were subject to an extended overnight incubation period in 1 × blocking buffer (1% BSA in PBT: 0.1% (v/v) Tween 20 in DPBS). The mouse anti-DIG biotin-conjugated antibody (Jackson ImmunoResearch Laboratories Inc. #200-062-(156) was prepared in a 1:200 dilution in blocking buffer and samples were incubated for 1.5 h. The final incubation in 100 μL of Tyramide working solution (Tyramide SuperBoost kit, ThermoFisher) was restricted to exactly 5 min, before incubating with an equal volume of stop reaction for 5 min.

Tissue samples were mounted in a drop of VECTASHIELD Mounting Medium with DAPI (Vector Laboratories) on a glass slide and imaged using the Revolve fluorescence microscope (ECHO). The FISH experiments were completed in at least three biological replicates that each included experimental (anti-sense) and control (sense) probes. Image acquisition settings were identical for each treatment (565 m s Texas red; 60 m s DAPI and 0 m s for TRANS/brightfield).

### Quantification and statistical analysis

All statistical analyses were performed in RStudio (version 4.3.3). The number of PCs to include in the snRNA-seq analysis was determined using a JackStraw test. Differential expression analyses were performed using a hurdle model in MAST and FDR correction used to account for multiple testing. RNA-FISH experiments were repeated independently three times and one representative image was shown for control and experimental probes. All additional data are available as Supplemental Data.

## Supplemental information

Document S1. Figures S1–S7 and Tables S1 and S2

Data S1. List of top 10 biomarker genes for all 22 cell clusters

Data S2. Differential expression of genes expressed in the SSCs in virgin vs. mated treatments

Data S3. GO enrichment of processes for genes differentially expressed in SSCs between virgin and mated females

Data S4. Differential expression of genes in the SSCs in saline vs. MAG-injected treatments

Data S5. GO enrichment of processes for genes differentially expressed in SSCs between saline- and MAG-injected females

Data S6. FlyphoneDB output of cell-cell signaling pathways in mated (Table 1) and virgin (Table 2) samples, with highly significant interactions (*p* < 0.001) highlighted in green
