# Supplementary material for: Transcriptional response of spermathecal secretory cells to mating and receipt of seminal fluid proteins in Aedes aegypti
Source: iScience. 2026 Jun 5;29(6):116301. doi: 10.1016/j.isci.2026.116301 (PMC13264323; doi:10.1016/j.isci.2026.116301)
Supplement: Document S1. Figures S1–S7 and Tables S1 and S2 [file mmc1.pdf]

iScience, Volume *29*

Supplemental information

Transcriptional response of spermathecal secretory cells to mating and receipt of seminal fluid proteins in *Aedes aegypti*

Claudia A.S. Wyer, Mehrnaz Afkhami, David A. Ellis, Sylvie Pitcher, I. Alexandra Amaro, Patrick K. Perish, Jake W. Angelico, Emilie M. Gray, Yasir H. Ahmed-Braimah, Mariana F. Wolfner, and Laura C. Harrington

Supplementary figures and tables

Supplementary Table 1. Top 10 biomarker genes for spermathecal secretory cell clusters

Biomarker rank	Gene ID	Gene name	% expression in SSCs	% expression in all other cells
1	LOC5573506	transcription factor vestigial	0.963	0.108
2	LOC5573818	Na[+]-driven anion exchanger 1	0.922	0.182
3	LOC5564562	LOC5564562	0.822	0.110
4	LOC5570922	Na[+]/H[+] hydrogen exchanger 2	0.901	0.197
5	LOC5577062	LOC5577062	0.988	0.310
6	LOC5575395	erythrocyte band 7 integral membrane protein	0.859	0.182
7	LOC5564265	LOC5564265	0.913	0.273
8	LOC5579397	LOC5579397	0.729	0.115
9	LOC5570905	cGMP-dependent 3',5'-cyclic phosphodiesterase	0.817	0.205
10	LOC5565190	Ras-like protein A	0.798	0.193

Supplementary Table 2. Oligonucleotides used for *Ae. aegypti* gene amplification and fluorescent *in situ* hybridization probe synthesis.

Primer name	Sequence	Amplicon size (bp)	T7 version of the primer
LOC557706 2 forward 1	GTCAGCTCAATAT GATCAGCAACC	587	**TAATACGACTCACTATAGGG**GTCAGCTCAATATGATCA GCAACC
LOC557706 2 reverse 1	CTGCCGGATCAGT TCTTCCAT		**TAATACGACTCACTATAGGG**CTGCCGGATCAGTTCTT CCAT

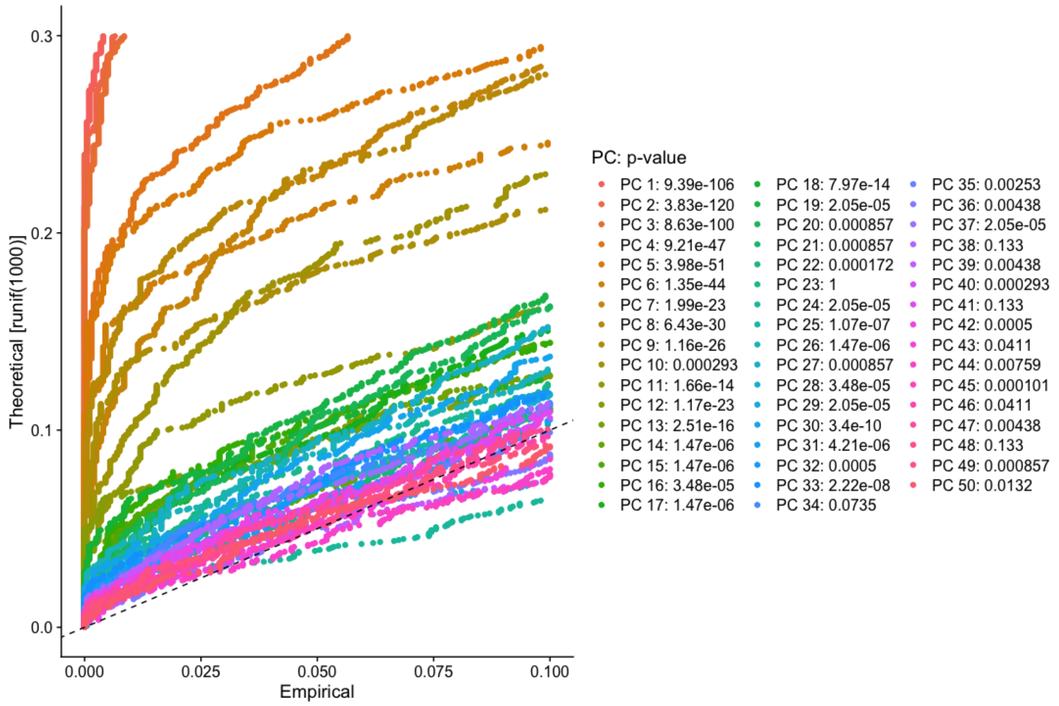

Supplementary Figure 1. Jackstraw analysis of principal component significance. Related to Figure 2

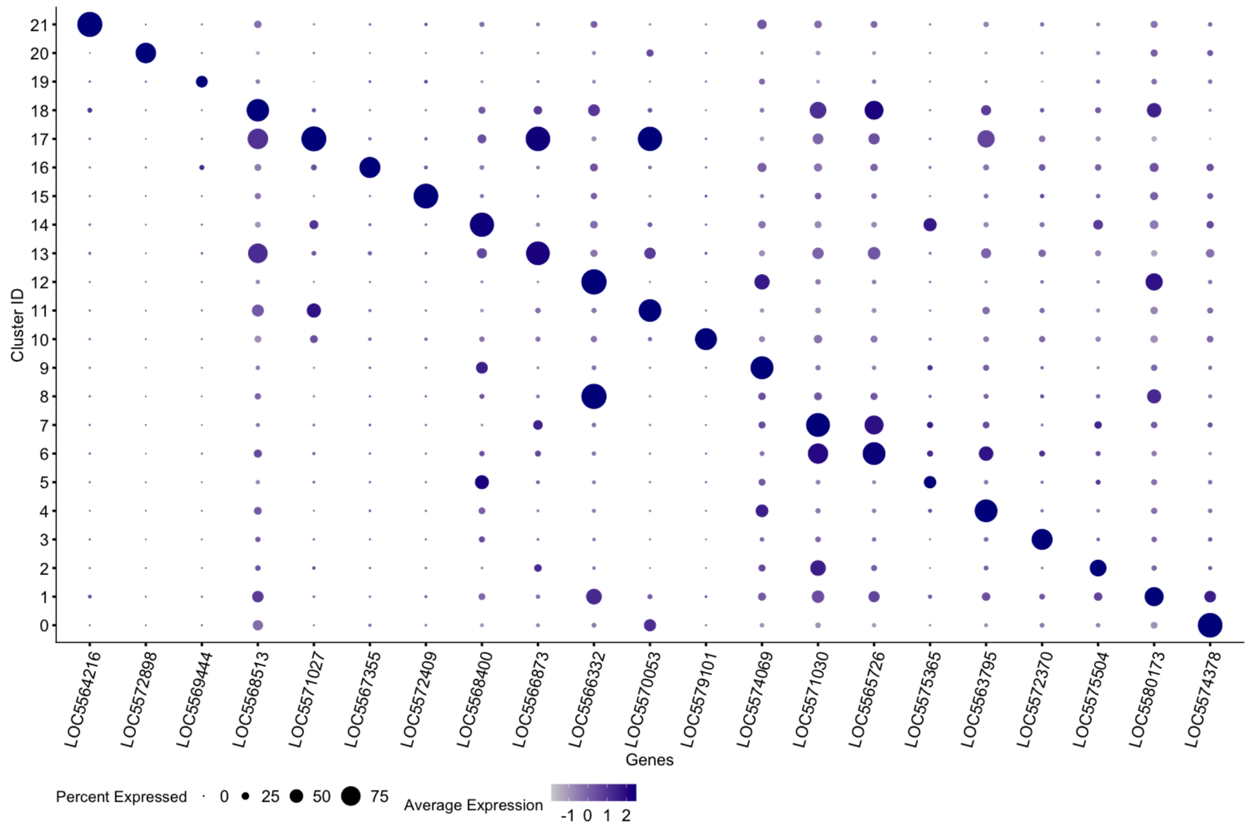

Supplementary Figure 2. Marker gene expression across cell clusters. Dot color reflects the average gene expression level and size reflects the percentage of expressing cells in that cluster. Related to Figure 2.

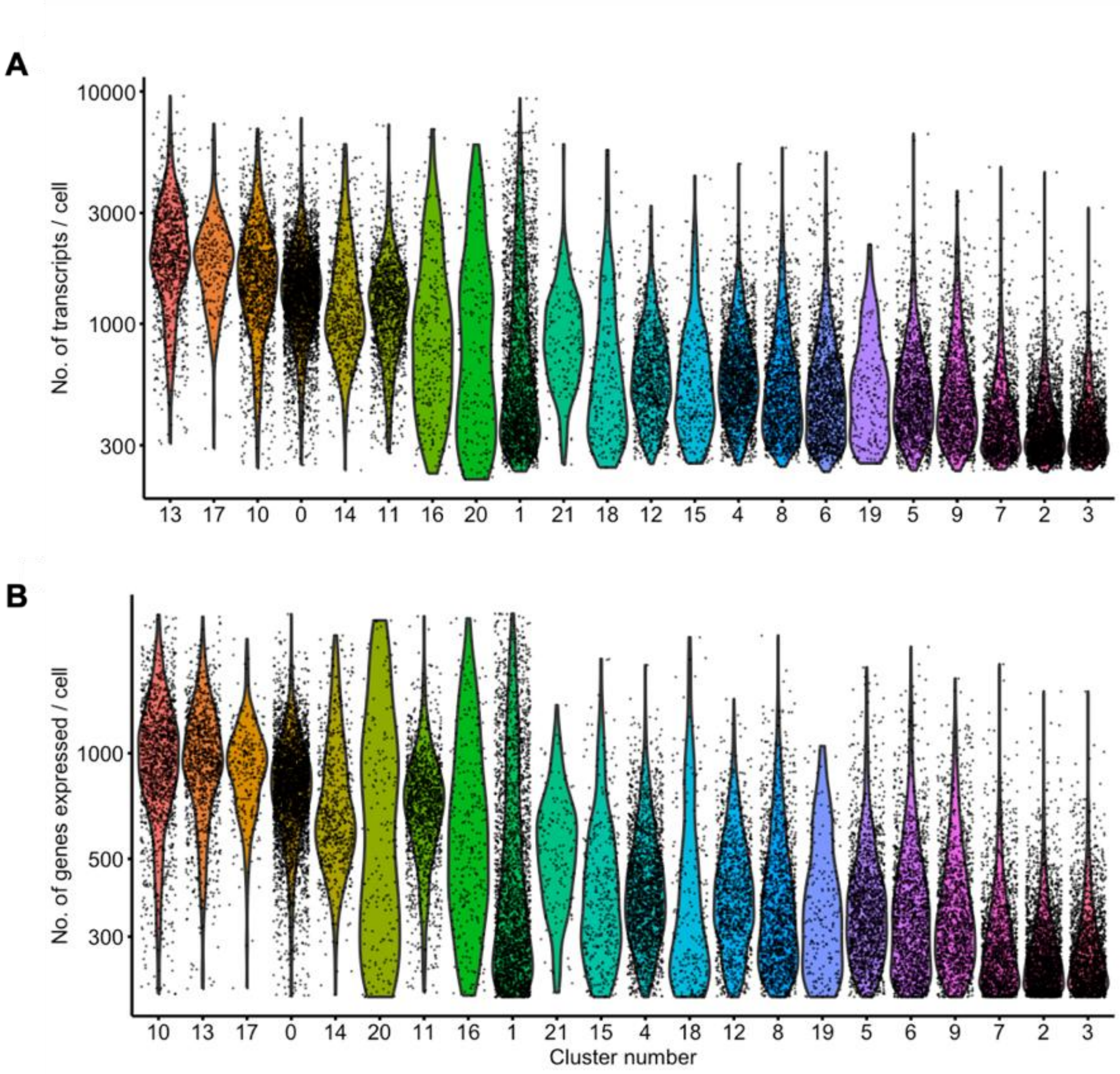

Supplementary Figure 3. Cluster-specific cell features. A) Total number of RNA transcripts detected for each cell. Clusters ranked from highest to lowest number of RNA transcripts per cell **B)** Total number of genes expressed for each cell. Clusters ranked from highest to lowest number of RNA transcripts per cell. For each plot, features values are plotted on a log scale. Related to Figure 2.

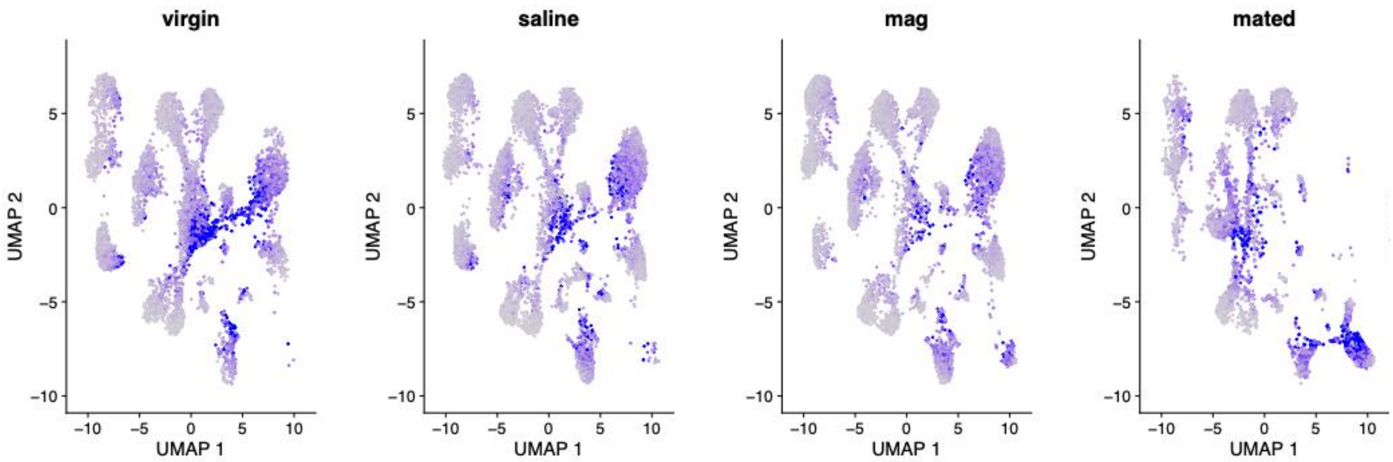

Supplementary Figure 4. Signal peptide analysis. UMAP of all filtered cells from snRNA-seq dataset. Individual cells colored according to the number of genes they express that contain a signal peptide sequence. Darker purple indicates a greater number of signal peptide genes expressed. Related to Figure 2.

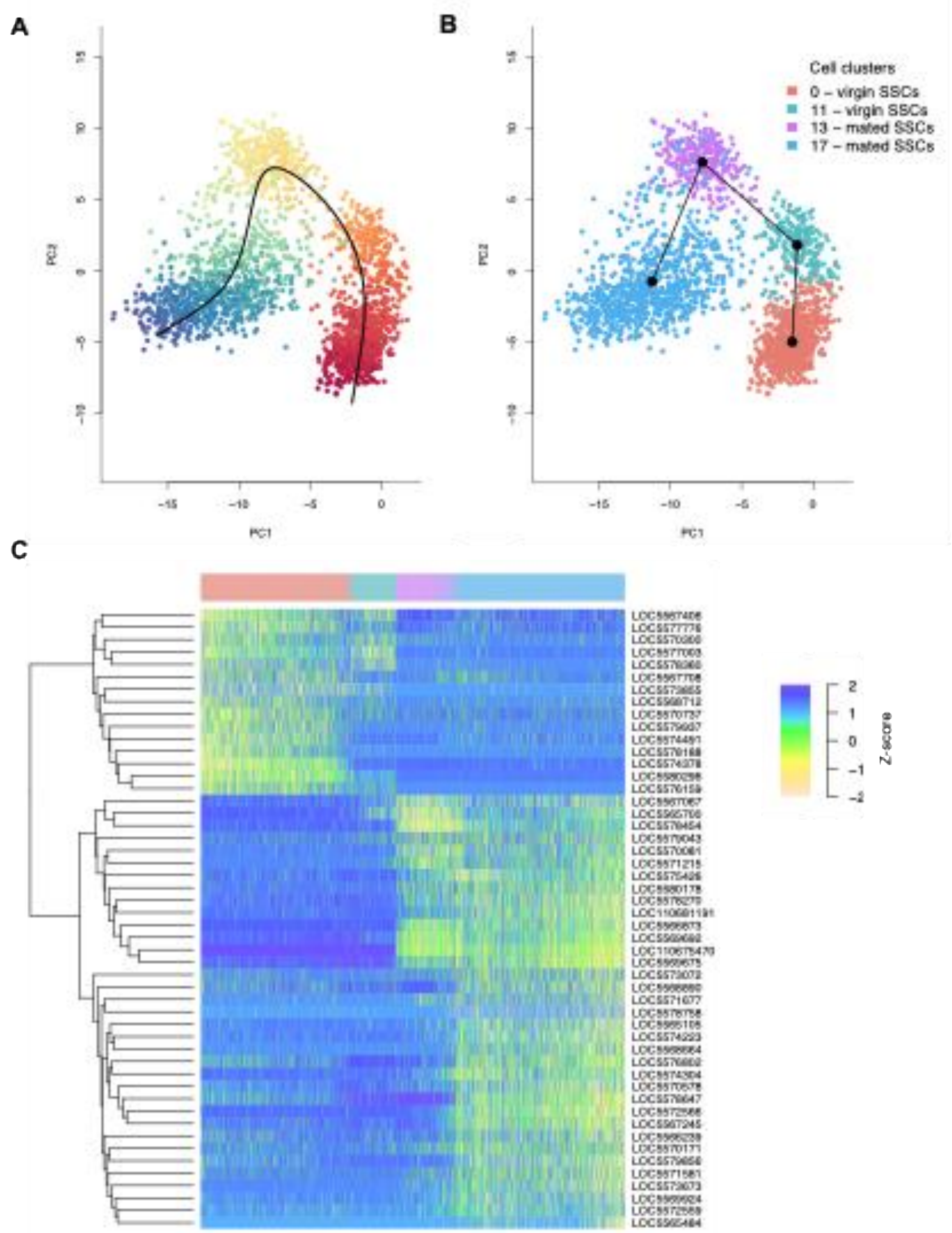

Supplementary Figure 5. Slingshot trajectory inference of SSC populations. A) Virgin and Mated SSCs projected into the first two principal components (PC1 and PC2), with each cell colored by its Slingshot pseudotime value along Lineage 1 and inferred Slingshot lineage curve overlayed. **B)** Cluster-level view of slingshot trajectory. **C)** Heatmap of the normalized (z-score) expression values for the 50 most significant genes, where rows correspond to genes and columns to cells ordered by pseudotime clusters**.** Related to Figure 3.

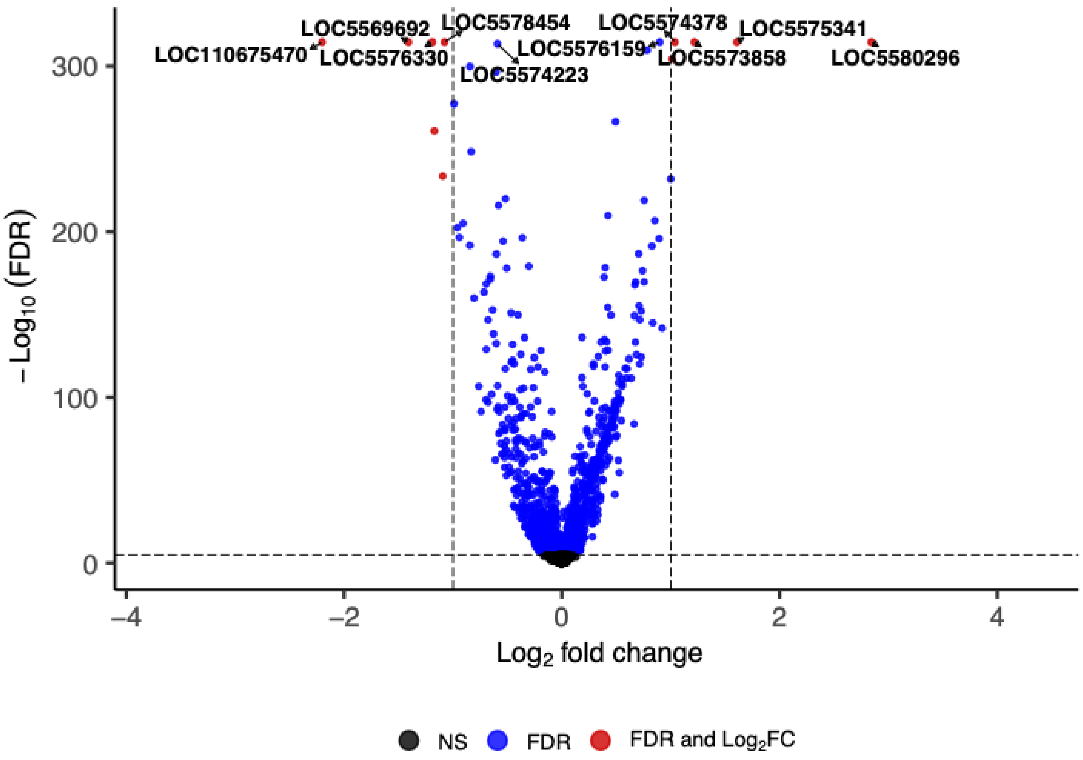

Supplementary Figure 6. Differential expression analysis of all cells between virgin and mated females. Quantitative changes in gene expression level between all filtered cells from mated versus virgin females. Significant genes based on FDR cutoff denoted by blue dots, significant genes based on FDR cutoff and Log_2 fold change greater than 1 denoted by red dots. FDR and Log_2 fold change cut offs demarcated with dotted lines. Related to Figure 5.

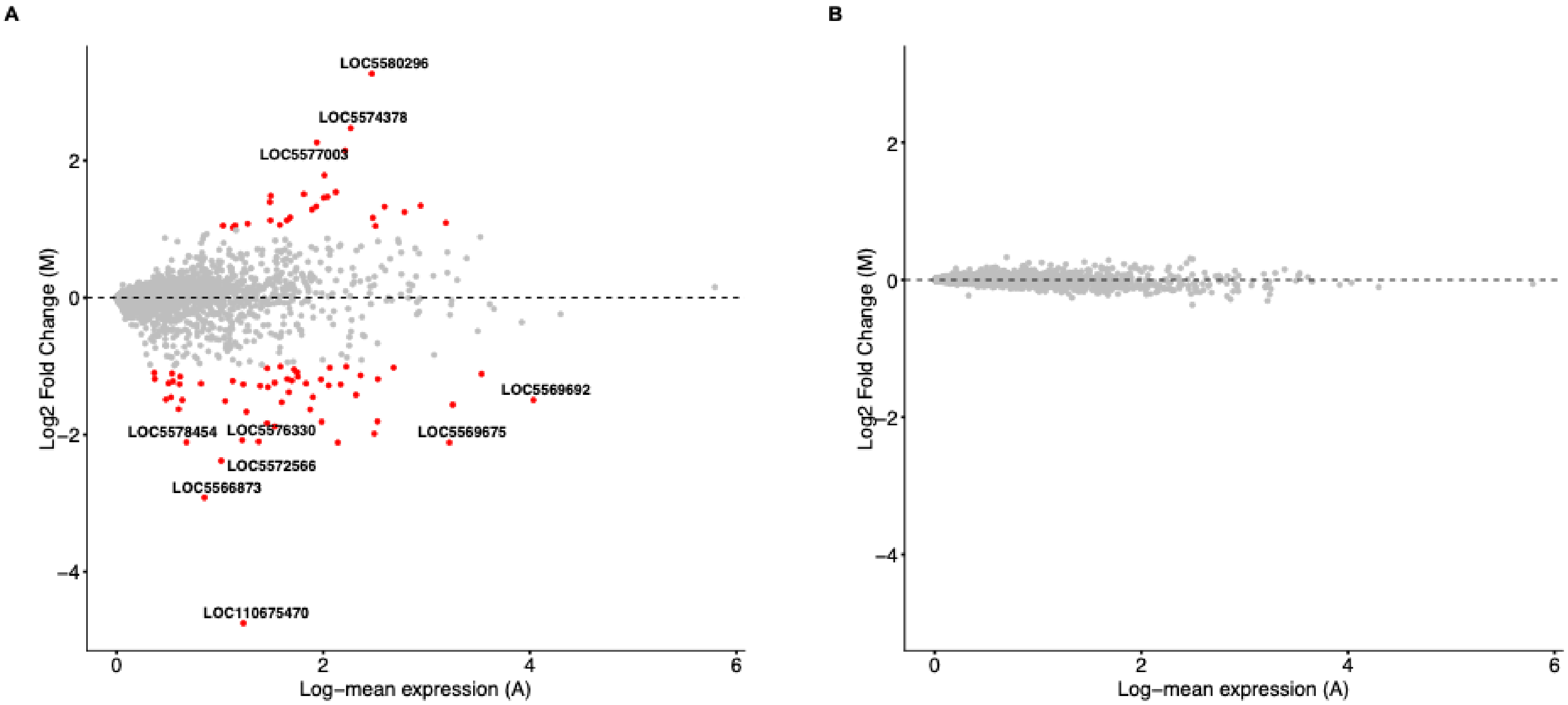

Supplementary Figure 7. MA plots of differential gene expression in SSC populations. A) Differential expression between virgin and mated females. **B)** Differential expression between MAG-injected and saline-injected females. In both panels, each point represents a gene plotted as $\log_2$ fold change (M) versus mean log-expression (A). Genes meeting significance thresholds (FDR < 0.01 and $\log_2$ fold change > 1) are highlighted in red, while non-significant genes are shown in grey. The top 10 differentially expressed genes are labeled. The horizontal line at M = 0 indicates no change in expression between conditions. Related to Figure 5.
